# Management of Refractory Cancer Pain with Intrathecal Drug Delivery and Spinal Cord Stimulation

**DOI:** 10.1089/pmr.2023.0089

**Published:** 2024-08-02

**Authors:** Evgeny Bulat, Jason E. Crowther, Vikram Chakravarthy, Ilya Laufer, Ori Barzilai, Amitabh Gulati

**Affiliations:** ^1^ Department of Anesthesiology, Perioperative and Pain Medicine, Brigham and Women’s Hospital, Boston, Massachusetts, USA.; ^2^ Department of Anesthesia, Critical Care & Pain Medicine, Massachusetts General Hospital, Boston, Massachusetts, USA.; ^3^ Department of Neurosurgery, The Ohio State University Wexner Medical Center, Columbus, Ohio, USA.; ^4^ Department of Neurosurgery, New York University, New York, New York, USA.; ^5^ Department of Neurosurgery, Memorial Sloan Kettering Cancer Center, New York, New York, USA.; ^6^ Department of Anesthesiology and Critical Care Medicine, Memorial Sloan Kettering Cancer Center, New York, New York, USA.

**Keywords:** cancer pain, intrathecal pump, neuromodulation, opioids, spinal cord stimulator

## Abstract

**Background:**

Intrathecal pumps (ITPs) are indicated for refractory cancer pain and decrease systemic opioid requirements. While not yet indicated for cancer pain, spinal cord stimulators (SCSs) are used off-label for cancer pain, with increasing evidence of their efficacy.

**Materials and Methods:**

A retrospective chart review was conducted of patients who underwent both ITP and at least SCS trial for cancer pain. Primary outcomes were pain numeric rating scale (NRS) and daily morphine equivalents (MEQs).

**Results:**

Seventeen patients were identified. Both ITP and SCS were associated with significant decreases in pain ratings at the 3-month follow-up, but this decrease became nonsignificant subsequently. ITP, but not SCS, was associated with a significant decrease in MEQ.

**Conclusions:**

ITP and SCS may both provide efficacy for cancer pain, but the opioid-sparing effects of SCS may be limited. ITP and SCS may potentially be complementary in their ability to provide relief from cancer-related pain.

## Introduction

Although cancer includes a variety of different pathologies, one commonality of many is the development of pain. Half of cancer patients report suffering from pain, and one-third report suffering from moderate-to-severe pain.^1^ The prevalence of pain increases with disease severity, but even 30–40% of patients who have undergone curative treatments continue to experience pain.^2,3^ Although medical management of cancer pain has improved, there is still a notable fraction of cancer patients with inadequately controlled chronic pain despite maximal medical management.^4^ In cases in which medical management is insufficient, interventions—including the implantation of devices for pain control—are an option.

Intrathecal pumps (ITPs) have demonstrated efficacy for pain, and are indicated for the treatment of refractory cancer pain. Advantages of ITPs include the ability to deliver opioid medications directly to the spinal cord, thus reducing the dose of systemic opioids and accompanying side effects, and to deliver medications that often cannot be given safely, or at all, systemically, such as local anesthetics and ziconotide.^5^ Disadvantages of ITPs include the necessity for frequent pump refills and potential complications, including accidental overdose or abrupt withdrawal with pump failure.^6,7^ ITPs are considered to be most appropriate for cancer patients with a life expectancy of 6 months or greater based on cost effectiveness, compared with other options available to patients with a worse prognosis.^8^

Spinal cord stimulators (SCSs) likewise modulate pain via direct interaction with the spinal cord but do so through electrical stimulation. Although SCSs are indicated for a variety of pain conditions, they are not yet indicated for the treatment of cancer pain, although many physicians have used them off-label for such patients. Research into SCSs for the treatment of cancer pain has been hampered for decades by the lack of MRI-conditional SCS systems, which were only developed in the last two decades. Before this, implantation of an SCS would preclude a cancer patient from having an MRI in the future, unless the SCS was surgically removed. Although SCS research in cancer pain is now becoming more common, so far the evidence for efficacy of SCS in the treatment of cancer pain is much more limited compared with the evidence for intrathecal (IT) drug delivery.^9^ Implantation of a SCS most commonly is a two-step process in which the patient first has percutaneous placement of temporary electrical leads with an external battery. After a one-week trial period, the leads are removed in a clinic setting. If the patients report significant pain relief during the trial period, they can subsequently elect to undergo implantation of permanent SCS leads and subcutaneous placement of a pulse generator/battery.

The current study is a retrospective review of select cancer patients treated at a single, large academic center who underwent both ITP and SCS implantation at some point during the treatment of their chronic cancer-related pain. The goal was to provide preliminary evidence as to whether both technologies may provide similar efficacy, and whether their unique mechanisms may be complementary.

## Methods

The current study was a retrospective cohort study at a quaternary cancer center. Patients were included if they were at least 18 years old, being treated for cancer pain (resulting either directly from cancer or as a result of cancer treatment), and underwent both ITP and *at least* an SCS trial lead placement, in either order, between 2003 and 2021. This study was approved by the Memorial Sloan Kettering Cancer Center Institutional Review Board (MSKCC IRB #17–537) and supported by the NIH Core Grant P30 CA008748.

All data were obtained through retrospective chart review of outpatient clinic and inpatient records. Patients’ baseline demographics, pain level, and pain management regimen were obtained within one month before each intervention (see Table 1). For the SCS trial leads, patients were assessed immediately before trial lead removal. For the SCS implant and ITP, patients were assessed within approximately one month, three months, and 1 year post-intervention. For both SCS trials and implants, paresthesia modalities were used to target the primary area of pain.

**Table 1.** Patient cohort demographics

Age at SCS trial	Years	Order of intervention	N (%)	Primary cancer type	N
Median	53.4	ITP first	5 (29)	Thyroid	1
Interquartile range (IQR)	23.6	SCS first	12 (71)	Parotid	1
Sex	***n* (%)**			Lung	1
Male	8 (47)	**Pain origin**	***n* (%)**	Breast	2
Female	9 (53)	Primary tumor	9 (53)	Pancreatic	2
Pain Location	***n***	Metastatic tumor	3 (18)	Renal	2
Chest	2	Postoperative	5 (29)	Prostate	1
Back	3			Testicular	1
Abdominal	2	**Pain type**	***n* (%)**	Endometrial	1
Pelvic	1	Nociceptive	3 (18)	Nerve	1
Extremity	6	Neuropathic	7 (41)	Colorectal	2
Multiple locations	3	Mixed	7 (41)	Sarcoma	2

IQR, interquartile range; ITP, intrathecal pump; SCS, spinal cord stimulator.

Primary outcomes were patient-reported average pain scores on the numeric rating scale (NRS) and systemic daily oral morphine equivalents (MEQ_S_) consumed. MEQ_S_ comprised oral, intravenous, transdermal, and sublingual opioids. IT opioids were separately tracked in daily IT morphine equivalents (MEQ_I_). Procedure-related complications were also recorded.

Nonparametric statistical analysis was performed, with alpha set to <0.05 for statistical significance.

## Results

Seventeen patients satisfied the study inclusion criteria. One patient had an ITP placed several years previously at another institution and not all records were available, while the remaining patients had both procedures performed at MSKCC. Of the patients, 12 (71%) had pain from active cancer and the remainder had pain as a result of cancer treatment. Extremity pain constituted the most common symptomatology, 82% had either mixed or neuropathic pain, and a variety of cancer types were represented. While most patients eventually underwent ITP after SCS, almost 30% underwent SCS after ITP. Seven patients (41%) underwent SCS implant after trial leads. The median time between SCS trial leads and ITP, or vice versa by order, was 132 days. Of the 12 patients who underwent SCS trial lead placement before ITP placement, 75% did not have permanent SCS implantation and instead opted for ITP implantation. Of these nine patients, six had failed SCS trials (no clinically significant improvement in pain) and three chose ITP because they hoped it would provide better pain control than SCS. There were six patients lost to follow-up due to mortality. Patients who underwent SCS trials were not included in the subsequent follow-up analysis if they did not undergo implant or, in the case of one patient, had an SCS implant but stopped using the SCS due to loss of efficacy.

Several complications were reported both for ITP and SCS implants. Two patients had SCS lead migrations requiring surgical revision, and 1 patient reported a significant loss of efficacy by 1 year postimplant. Two patients had ITP catheter tip migrations requiring surgical revision, and one patient reported a significant postdural puncture headache that was managed with an epidural blood patch. No significant infections were reported requiring explant for either ITP or SCS implants.

SCS implant and ITP were both associated with a two-point reduction by NRS (ΔNRS) within three months post-intervention (see Table 2). At 12–18 months, no significant ΔNRS was found for either intervention. ITP was associated with a significant change of MEQ_S_, or ΔMEQ_S_, of −126 to 130 mg within three months, but not at 18 months (Table 2). Neither SCS trial nor implant led to significant ΔMEQ at any follow-up.

**Table 2.** Primary outcomes of pain (NRS) and daily morphine equivalents (MEQ) across interventions

	Median follow-up (days)	Median (IQR) change by NRS	*p* value	Median (IQR) change by MEQ_S_ (mg)	*p* value	n
ITP						
1st follow-up	23	−2 (3.3)	**0.003**	−130 (313)	**0.008**	16
2nd follow-up	96	−2 (1.8)	**0.02**	−126 (188)	**0.008**	15
3rd follow-up	560	−0.5 (1.8)	0.2	−61 (386)	0.42	10
SCS trial	6	−1 (3.5)	**0.03**	0 (170)	0.55	14
SCS implant						
1st follow-up	29	−2 (2)	0.15	−162 (952)	0.2	7
2nd follow-up	85	−2 (1.5)	**0.016**	0 (3860)	0.66	7
3rd follow-up	352	+1 (3)	0.71	+170 (170)	1	3

IQR, interquartile range; ITP, intrathecal pump; SCS, spinal cord stimulator.

Significance for bold- *P*-value.

IT opioids were tracked after either ITP or SCS implant in patients who already underwent ITP (see Table 3). Only continuous, nonpatient-administered, doses were included. After ITP, there was a consistent increase in daily IT opioids at each follow-up. Following SCS implant in patients with ITP, no consistent increase in IT opioids was found.

**Table 3.** Intrathecal daily morphine equivalent dose (iMEQ) across interventions

	Median follow-up (days)	Median (IQR) MEQ_I_ (mg)	*p* value 1	*p* value 2	*p* value 3
ITP					
Baseline	—	—	—	—	
1st follow-up	23	2.3 (5.7)	**<0.001**		
2nd follow-up	96	2.7 (8.6)		**0.010**	
3rd follow-up	560	4.4 (6.1)			**0.004**
SCS trial	6	24.4 (10)	—	—	—
SCS implant					
Baseline	—	10 (12.8)	0.79		
1st follow-up	29	20 (21.2)		0.21	
2nd follow-up	85	29 (6.5)			0.31
3rd follow-up	—	—	—	—	—

IQR, interquartile range; ITP, intrathecal pump; SCS, spinal cord stimulator.

Significance for bold- *P*-value.

## Discussion

IT drug delivery has demonstrated efficacy in the management of refractory pain in cancer patients, whereas research on the use of spinal cord stimulation for palliative management has only more recently begun, in large part, due to its historical incompatibility with MRI. There is a growing body of evidence, primarily retrospective evidence at this time, that at least paresthesia-mode SCS may be beneficial for cancer pain.^9^

Our findings for patients who underwent both interventions for cancer pain suggest that for at least three months post-intervention, SCS may provide clinically significant symptomatic pain relief. Both ITP and SCS had diminished efficacy by 12–18 months, which conflicts with prior findings of a sustained response from ITP after 12 months,^10^ but this discrepancy may be due to loss to follow-up (primarily due to patient mortality) in an already small study cohort, as well as potential selection bias toward highly refractory cancer pain.

Our findings replicated previous literature documenting ITP’s opioid-sparing effect on systemic opioids. On the contrary, we did not find any opioid-sparing effect with SCS, which does fit with other findings in cancer patients.^11^ It is unclear why this effect did not persist for 18 months in the case of ITP, but this could also be due to the cohort size and follow-up issues discussed previously.

The sample size in the current study is not large enough for us to definitively compare SCS and ITP by efficacy in terms of pain relief for cancer patients with refractory pain. It is interesting to note that patients appeared to have improved pain control with both interventions, regardless of which intervention was performed first. Although speculative, it is perhaps suggestive that the two interventions modulate pain in independent and possibly complementary ways. However, when patients were given an initial choice between the two treatment modalities, and underwent a trial SCS placement first, the majority of patients chose to subsequently have an ITP placed instead of a permanent SCS. For some patients, this was because they had no clinically significant improvement in pain, but it appeared for others that they did not have as significant an improvement in pain as they expected to have from an ITP.

ITPs provide medication directly to the central nervous system, and medications are commonly individual opioids, local anesthetics, or other adjuvants (e.g., clonidine, ziconotide) or a combination of these medications. The opioid medications are hypothesized to improve pain control by increasing descending inhibitory signals, whereas the local anesthetics, although potentially affecting all neural fibers, primarily affect the smaller and unmyelinated fibers, which transmit pain signals.

Paresthesia-mode SCS is hypothesized to operate by a separate mechanism, in line with the gate-control theory of pain, which postulates that increasing activation of non-nociceptive, sensory stimulation will inhibit the transmission of nociceptive stimulation via inhibitory interneurons.^12^ Because of their independent mechanisms, ITP and SCS each could potentially contribute to improved pain control in a complementary manner. The vast majority of research in spinal cord stimulation, including in the cancer patient population, has involved paresthesia-mode SCS. This stimulation mode is now being replaced in the clinical setting by other modes of stimulation, including high-frequency and burst stimulation. There is less literature currently on these newer modes of stimulation, but there is suggestion that these may have additional mechanisms to reduce pain, such as by reducing inflammatory cytokines in the spinal cord to reduce central sensitivity to pain stimuli.^13^

In the current study, we sought to compare spinal cord stimulation and IT drug delivery for the management of chronic cancer pain in a group of patients who had undergone treatment with both modalities. As a whole, these patients did appear to derive benefit from both treatment modalities. Although it was not possible to draw any definitive conclusions about the relative efficacy of these two treatment modalities, we did find more consistent evidence for IT drug delivery benefiting patients both in terms of pain and systemic opioid reduction. Along with the greater body of evidence in the literature supporting the use of IT drug delivery for the treatment of cancer pain, this overall lends greater support to ITPs over SCSs for the treatment of cancer pain. Future research, particularly with newer SCS systems and stimulation modalities, will hopefully elucidate their relative efficacy, and under which circumstances, spinal cord stimulation might be preferred over IT drug delivery for specific patients.

### Limitations

The current study has some limitations. By far the most important is a small cohort, which limits the interpretability of nonsignificant findings and the ability to perform adequately powered between- or subgroup analyses. The retrospective study design limits the data’s standardization and precision, as well as the availability of data related to function and overall quality of life. In addition, while our study design allows for some “within-patient” control, it does not control for disease progression over time. Finally, our specific findings may not apply to newer, paresthesia-free SCS modes.

## Notes

### Abbreviations Used

IT: Intrathecal
ITP: Intrathecal pump
MEQ: Morphine equivalents
NRS: Numeric rating scale
SCS: Spinal cord stimulator

## References

[1] Snijders RAH, Brom L, Theunissen M, van den Beuken-van Everdingen MHJ. Update on prevalence of pain in patients with cancer 2022: A systematic literature review and meta-analysis. Cancers (Basel). 2023;15(3):591. 36765547 10.3390/cancers15030591 PMC9913127

[2] van den Beuken-van Everdingen MH, de Rijke JM, Kessels AG, et al. Prevalence of pain in patients with cancer: A systematic review of the past 40 years. Ann Oncol 2007;18(9):1437–1449. 17355955 10.1093/annonc/mdm056

[3] Glare PA, Davies PS, Finlay E, et al. Pain in cancer survivors. J Clin Oncol 2014;32(16):1703–1711. 24799477 10.1200/JCO.2013.52.4629 PMC4031191

[4] Scarborough BM, Smith CB. Optimal pain management for patients with cancer in the modern era. CA Cancer J Clin 2018;68(3):182–196. 29603142 10.3322/caac.21453 PMC5980731

[5] Spiegel MA, Chen GH, Solla AC, et al. Evaluation of an intrathecal drug delivery protocol leads to rapid reduction of systemic opioids in the oncological population. J Palliat Med 2021;24(3):418–422. 32640912 10.1089/jpm.2020.0206 PMC8182653

[6] Smith TJ, Staats PS, Deer T, et al. Randomized clinical trial of an implantable drug delivery system compared with comprehensive medical management for refractory cancer pain: Impact on pain, drug-related toxicity, and survival. J Clin Oncol 2002;20(19):4040–4049. 12351602 10.1200/JCO.2002.02.118

[7] Delhaas EM, Huygen FJPM. Complications associated with intrathecal drug delivery systems. BJA Educ 2020;20(2):51–57. 33456930 10.1016/j.bjae.2019.11.002 PMC7807963

[8] Chou CZ, Hopkins TJ, Badiola I, et al. Top ten tips palliative care clinicians should know about interventional pain and procedures. J Palliat Med 2020;23(10):1386–1391. 32865443 10.1089/jpm.2020.0487

[9] Hagedorn JM, Pittelkow TP, Hunt CL, et al. Current perspectives on spinal cord stimulation for the treatment of cancer pain. J Pain Res 2020;13:3295–3305. 33324090 10.2147/JPR.S263857 PMC7732175

[10] Stearns LM, Abd-Elsayed A, Perruchoud C, et al. Intrathecal drug delivery systems for cancer pain: An analysis of a prospective, multicenter product surveillance registry. Anesth Analg 2020;130(2):289–297. 31567325 10.1213/ANE.0000000000004425 PMC6948791

[11] Crowther JE, Chen GH, Legler A, Gulati A. Spinal cord stimulation in the treatment of cancer pain: A retrospective review. Neuromodulation 2022;25(5):693–699. 35410770 10.1016/j.neurom.2022.01.023

[12] Melzack R, Wall PD. Pain mechanisms: A new theory. Science 1965;150(3699):971–979. 5320816 10.1126/science.150.3699.971

[13] Echeverria-Villalobos M, Mitchell J, Fiorda-Diaz J, Weaver T. Effects of dorsal column spinal cord stimulation on neuroinflammation: Revisiting molecular mechanisms and clinical outcomes on chronic lumbar/leg pain and failed back surgery syndrome. J Pain Res 2021;14:2337–2345. 34354373 10.2147/JPR.S309872 PMC8331196

